# Conceptualization of patient‐centered care in Latin America: A scoping review

**DOI:** 10.1111/hex.13797

**Published:** 2023-07-25

**Authors:** Anne Klimesch, Alejandra Martinez‐Pereira, Cheyenne Topf, Martin Härter, Isabelle Scholl, Paulina Bravo

**Affiliations:** ^1^ Department of Psychiatry and Psychotherapy University Medical Center Hamburg‐Eppendorf Hamburg Germany; ^2^ Department of Medical Psychology University Medical Center Hamburg‐Eppendorf Hamburg Germany; ^3^ School of Nursing Pontificia Universidad Católica de Chile Santiago Chile; ^4^ Instituto Oncológico Fundación Arturo López Pérez Santiago Chile

**Keywords:** family‐centered care, Latin America, patient‐centered care, person‐centered care

## Abstract

**Introduction:**

Patient‐centered care (PCC) has been declared as a desirable goal for health care in Latin American countries, but a coherent definition of what exactly PCC entails for clinical practice is missing. This article's aim was to identify how PCC is conceptualized in Latin American countries.

**Methods:**

Scientific databases (MEDLINE, EMBASE, PsycINFO, CINAHL, Scielo, Scopus, Web of Science) and webpages of the ministries of health were searched, and experts were contacted for suggestions of literature. References were included if they contained one of a range of a priori defined keywords related to PCC in the title, were published between 2006 and 2021, and were carried out in or concerned Latin America. Definitions of PCC were extracted from the included articles and analyzed using deductive and inductive coding. Deductive coding was based on the integrative model of patient‐centeredness, which unites the definitions of PCC in the international literature (mainly North America and Europe) and proposes 16 dimensions describing PCC.

**Results:**

Thirty‐two articles were included in the analysis and about half of them were from Brazil. Numerous similarities were found between the integrative model of patient‐centeredness and the definitions of PCC given in the selected literature. The dimensions of the integrative model of patient‐centeredness that were least and most prominent in the literature were physical support and patient information, respectively. A differentiation between PCC and family‐centered care (FCC) was observed. Definitions of PCC and FCC as well as their cited references were diverse.

**Conclusion:**

A considerable overlap between the conceptualization of PCC in Latin America and the integrative model of patient‐centeredness has been identified. However, there are substantial differences between countries in Latin America regarding the emphasis of research on PCC versus FCC and diverse conceptualizations of PCC and FCC exist.

**Patient Contribution:**

This scoping review takes the patient's perspective based on the integrative model of patient‐centeredness. Due to the study being a review, no patients, neither caregivers, nor members of the public, were involved.

## 1 INTRODUCTION

In an effort to improve population health, the global community has worked towards the development and advancement of health care systems around the world.^1^ Health outcomes have globally improved, leading to an aging population over the past decades. An aging population brings about novel challenges to health care systems, for example, increasing prevalence of chronic noncommunicable diseases.^2^ These developments were complemented by a retraction from the paternalistic approach to health care and the emergence of alternative concepts as patient‐centered care (PCC). In a paternalistic health care setting, the health care professional (HCP) is an authority who applies objective criteria to determine the treatment plan and informs the patient about the chosen intervention.^3^ PCC proposes a shift towards balanced power in the relationship between HCP and patient, towards patient empowerment, active participation of the patient in the health care process, as well as a focus on individual patient needs, values, and preferences.^4^, ^5^

Arguments in favor of PCC are of ethical, moral, and scientific nature. To treat all patients equally, respectfully, and recognize their autonomy are standards of medical ethics^6^ and promoted by PCC.^5^ It is emphasized that essentials to health care are, among others, cultural appropriateness, provision of information, recognition of individual circumstances and needs, and access to care without discrimination.^7^ These standards are supposed to decrease inequalities in access to health care. Research suggests an association between aspects of PCC and positive patient outcomes, for example, health status,^8^ treatment adherence,^9^ costs,^10^ health behavior,^11^ social support, quality of medical decisions,^12^ and self‐rated health.^13^ Thus, diverse lines of argumentation suggest PCC to be a desirable process and outcome in health care.

The increasing number of scientific publications on PCC has brought about diverse definitions of PCC in the international literature. Scholl et al.^5^ saw a need for a coherent conceptualization of PCC which would provide common ground for future scientific and health policy work on PCC. To address this need, Scholl et al.^5^ developed the integrative model of patient‐centeredness (henceforth “integrative model”) by a systematic synthesis of diverse definitions of PCC described in the international literature, mainly from North America and Europe, but none from Latin America. The model proposes 15 dimensions of PCC (Supporting Information: Appendix S1) and has since been used in research on PCC, for example, in the development of a patient‐reported experience measure of PCC^14^ and came to close a gap in the international conceptualization of PCC.^15^, ^16^, ^17^

Research on and implementation of PCC have not been uniform around the world. PCC has been widely described and investigated in the global north.^18^ In contrast, in regions where accessibility to health care and social inequalities remain an issue, as in Latin America,^19^ there has been comparably little research on PCC. The socioeconomic, political, and economic structures of Latin American countries are diverse.^20^ After the end of colonialization, military dictatorships undermining human rights were implemented in many countries, which lead to socioeconomic and health inequalities in Latin America.^20^ Social movements achieved the restatement of civilian rule in some countries. These political changes as well as economic growth were precursors for health system reforms that have been implemented in Latin American countries to achieve universal health coverage and decrease poverty over the past decades.^20^ For example, in Chile, health system reforms have led to a health coverage of about 95%.^21^ However, health systems in many Latin American countries constitute a mixture of the public and a private sector, which promotes health inequalities and could enhance the continuation of a paternalistic style in health care.^22^ In 2018, a survey conducted by the Organization for Economic Co‐operation and Development (OECD) indicated that the spread and degree of health care coverage are less uniform in 21 Latin American countries in comparison to other OECD countries.^23^

With regard to PCC, in 2003, the Pan American Health Organization declared strategies to implement the principles of “equity, solidarity, and the right to the highest possible standard of health” in Latin American health care systems.^24^ In line with this, access to care has successfully been improved in Mexico by the introduction of a program, which provides affordable health care to uninsured individuals.^25^ Another example is Chile, where PCC has been declared as one of the fundamental principles of the health system in 2006.^26^ Thus, health policymakers in Latin America have recognized the need for PCC and claimed the intention to establish PCC in routine care.^24^, ^25^, ^26^ In 2016, the OECD implemented a *Latin America and the Caribbean Network of Health Systems* to “identify effective policies to ensure the financial sustainability of health systems” (OECD‐LAC Regional Policy Networks).

Latin American research on PCC shows little coherence in the conceptualization of PCC. For example, Guanais et al.^13^ conducted a secondary analysis of a public opinion survey on the health care system which had been conducted in six Latin American countries. They chose the following variables as being related to PCC for analysis: contact with primary care clinic (access), time spent with HCP, patient‐HCP communication, technical quality and problem solving, and health care coordination. In contrast, in another analysis of patient‐reported experience with health care in four Latin American countries, variables that were considered to be associated with PCC were easy access, coordinated care, good HCP–patient communication, provision of health‐related information and education, and emotional support.^27^ The difference between variables considered to be associated with PCC in the two studies represents variations in the conceptualization of PCC in Latin American research. Moreover, it is unclear how the concept of PCC has evolved in Latin America. As Scholl et al.^5^ have recognized before, a clear concept describing PCC is necessary to compare research results and to implement PCC.

Despite the advances in health care and research on PCC, researchers from Chile have shown that thorough implementation of PCC is still missing.^28^ Patients reported a lack of opportunities for active participation in medical decision‐making in primary care and a disbalance in the distribution of power between HCPs and patients. Moreover, patient satisfaction with public health care significantly decreased from 2010 to 2015.^29^ In a survey carried out in six Latin American countries, more than 80% of participants indicated that their health care system required substantial changes.^30^ One main issue recorded by these surveys was access to care, which is an aspect of PCC. One reason for the lack of implementation of PCC in practice could be that clear guidelines on how to put patients at the center of care and let them participate in decision‐making are missing.^31^ In line with that, Bravo et al.^32^ suggest that a clear operationalization of PCC in the Latin American context is needed. Thus, the aim of this scoping review is to analyze how PCC is conceptualized in Latin America.

To date, there is no coherent definition of PCC in Latin America. Therefore, the declared aim to implement PCC in Latin America can hardly be achieved. The research question of this review is: How does the conceptualization of PCC in Latin America differ from the integrative model? The integrative model will be used as a point of reference because it is internationally established and based on international literature except in Latin America. It is thus suited for comparison and potential extension by the results of the scoping review. The comparison fosters the development of one joint conceptualization of PCC in Latin America and internationally. This enables comparability and therefore also communication and collaboration in research as well as in implementation. The result of this review can thus support the declared aim to implement PCC in Latin America.

## 2 METHODS

To address the research question, a scoping review^33^ was conducted following the framework of Peters et al.^33^

### 2.1 Search strategy

We developed a protocol following Peters et al.^33^ and defined the population as the general population in Latin America, the concept as PCC, and the context as health care in general. The protocol can be received from the authors upon request. Two reviewers (A. K. and A. M.) conducted the electronic literature searches in MEDLINE, EMBASE, PsycINFO, CINAHL, Scopus, Scielo, and Web of Science between April and May 2021. Articles were included if they were published between January 2006 and December 2021. We limited the search to 15 years, considering milestones in Latin American countries on the implementation of PCC (e.g., health reform in Chile in 2006). Articles were included if published in the regions' official languages: English, Spanish, French, and Portuguese. We carried out a secondary literature search by asking Latin American experts in PCC for relevant references. Finally, a gray literature search was conducted on the webpages of the ministries of health of each country in Latin America.

### 2.2 Eligibility criteria

In the initial search, we included articles that contained one of the following terms in the title and abstract: patient‐centered, person‐centered, family‐centered (each with four spelling variations) and patient‐focused (with two spelling variations). In addition, titles and abstracts of the records had to contain either the term Latin America or the name of one of the 27 Latin American countries. In addition to scientific articles, opinion articles, discussion articles, editorials, letters to the editor, statements, and books were included. There was no exclusion criterion regarding the study design or setting. During the title‐abstract screening, studies and other records were excluded if they had not been carried out in Latin America or did not discuss their major content in the context of a Latin American country. Records were excluded if they did not discuss the key term, upon which they had been included in the initial search, in the context of health care. In the full‐text screening, records were only maintained, if they contained a definition of the key term.

### 2.3 Study selection process

The identified records were imported into Endnote X9^34^ and duplicates (1465) were removed. Two reviewers (A. K. and A. M.) conducted the title and abstract screening, and three reviewers (A. K., A. M., and C. T.) did the full‐text screening and data extraction. We randomly distributed the articles among the reviewers. Spanish articles were read only by two (A. M. and A. K.), and Portuguese articles were read by A. M. and reviewed by P. B. Each article was double‐screened and compared among the respective reviewers. The number of articles was balanced out between reviewers. Finally, A. M. and A. K. reviewed all the extracted information and codes and discussed discrepancies to reach an agreement. Doubts about terms and concepts were discussed by the team (A. K., A. M., I. S., and P. B.).

### 2.4 Data extraction

The following data were extracted using a data extraction sheet including country of publication, the description of the main concept, study design, data acquisition, sample characteristics, health setting, and the conclusion drawn by the respective paper regarding the main concept. As we suggest that one joint model of PCC based on international research is desirable, we used the integrative model by Scholl et al.^5^ for the analysis of conceptualizations of PCC in Latin America. For every paper, it was extracted regarding whether the 15 dimensions of the integrative model were mentioned. This was done by the deductive coding. Aspects related to PCC that were mentioned in the selected literature, but not covered by the integrative model were extracted separately. A. K. and E. C. discussed whether these were new dimensions or could be subsumed into one of the 15 dimensions of the model. The references provided for the definitions of PCC and family‐centered care (FCC) were analyzed regarding repetition in the sample and their origin.

### 2.5 Synthesis and analysis

To answer the research question of how the conceptualization of PCC in Latin America differs from the integrative model, the following descriptive information was analyzed for the selected literature: frequency of publication type, distribution of publication years per the central concept, frequency of publications per country in Latin America, and repetition of authors who published the included literature. The extracted main concepts were grouped based on content, and the resulting division was considered in all further analyses. To understand the origin of conceptualizations of PCC in the selected literature, the references provided for the definitions of PCC were analyzed with respect to their origin and repetition between articles. The results of the deductive coding regarding the 15 dimensions of the integrative model^5^ were analyzed by A. K. with respect to the occurrence and frequency of each dimension. We carried out a content analysis^35^ of the conceptual definitions of PCC and FCC, following these steps: (1) development of the research question; (2) selection of the categories of analysis; (3) collection of data in a predetermined coding agenda; (4) revision of categories and coding agenda into meaningful clusters (principles, activities, results); (5) final interpretation of the results. All analyses were done in Microsoft Excel.^36^

## 3 RESULTS

### 3.1 Descriptive information

**Figure 1:**
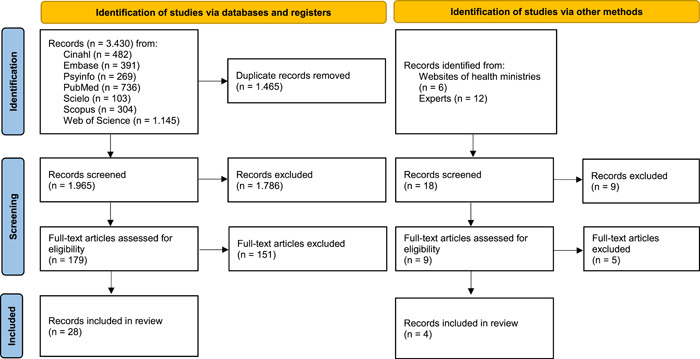
PRISMA 2020 flow diagram of study selection.

The initial electronic literature search identified 3430 articles (1465 duplicates). Based on the secondary search and gray literature search, 18 articles were added. After the full‐text screening, 32 articles were included in the analysis. For the PRISMA 2020 flow chart,^37^ see Figure 1.

**Figure 2:**
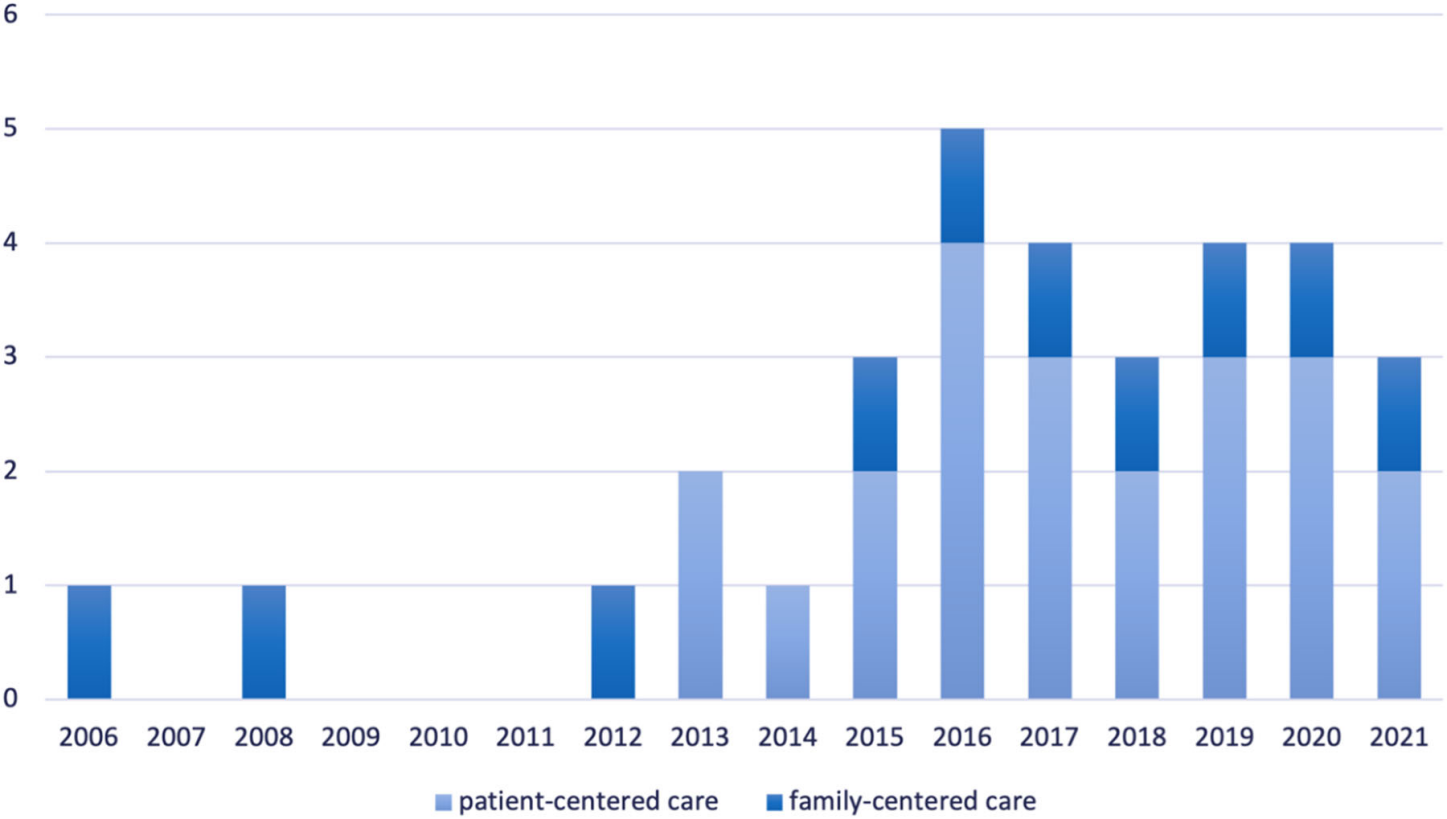
Year of publication distinguished by main concept of the articles (*n* = 32).

The reasons for excluding articles during the title and abstract screening and full‐text screening were that articles turned out to be from outside Latin America. For example, studies were included based on the search term “Mexico,” but were later identified as studies from New Mexico, USA. Similarly, studies on PCC for Latin American immigrants in the United States of America, published by authors from the United States of America, were excluded. Another article was excluded because the study was completely conducted in Spain, even though one coauthor was affiliated with an institution in Latin America. At least 18 articles were excluded for missing a definition of the main concept the article was discussing (e.g., PCC). Papers focusing on person‐centered research methods instead of health care were also excluded. Finally, articles on the person‐centered therapy developed by Carl Rogers were excluded because the articles took a therapeutic perspective on PCC, instead of a system‐based perspective, which is of interest for this study. The selected literature comprises 29 research papers,^13^, ^27^, ^28^, ^32^, ^38^, ^39^, ^40^, ^41^, ^42^, ^43^, ^44^, ^45^, ^46^, ^47^, ^48^, ^49^, ^50^, ^51^, ^52^, ^53^, ^54^, ^55^, ^56^, ^57^ two health policy documents,^26^, ^58^ and one conference abstract.^59^ Most studies (*n* = 27) were published between 2013 and 2021. For an overview of the publication year and the main concept of the included articles, see Figure 2.

Almost half of the articles (*n* = 15) were published by authors from Brazil,^38^, ^39^, ^41^, ^42^, ^44^, ^45^, ^47^, ^49^, ^50^, ^51^, ^53^, ^54^, ^55^, ^57^, ^60^ six were published by authors from Chile (two health policies,^26^, ^58^ four research articles^28^, ^32^, ^48^), and five by authors from Mexico (one conference abstract,^59^ four research articles^43^, ^56^, ^61^). Four papers were published by authors from several Latin American countries,^13^, ^27^, ^40^, ^62^ and one article was published by authors from Colombia^46^ and Honduras^63^ each. In the included literature on PCC, there were authors who repeatedly occurred, either as first‐, co‐authors, or last‐authors: Doubova, S. V. (5), Bravo, P. (3), Dois, A. (3), Martinez‐Vega, I.P. (2), Ministerio de Salud Chile (2). In the literature focused on FCC, each article was published by different authors.

### 3.2 Main concepts

In the selected literature, PCC was discussed using diverse terms. These terms were grouped into PCC and FCC categories, which will be referred to as main concepts in the following. In most articles (*n* = 22) the main concept was PCC. Twenty‐four different terms were used to refer to this main concept. One article described PCC in the context of the Biomedical Model of Care.^59^ FCC was the main concept of 10 articles and within these, four different terms were used to refer to FCC. For an overview of all the terms used to refer to the main concepts in the selected literature, see Table 1.

**Table 1:** Main concept of the articles and their frequency (*n* = 32) are thematically ordered.

	Main concept	Count	Main concept	Count
	Patient‐centered/‐centered care	9	Family‐centered care	6
Associated terms *(frequency if other than 1*)	Person‐centered care (3) − Patient‐centered clinical model − Patient‐centered orientation for health care − Patient‐centered clinical method − Patient‐centered primary care − Patient‐centered medical practice − Patient‐centredness − Patient/family‐centered care	9	− Family‐centered care model − Family‐centered practice − Family‐centeredness	3
*Associated Spanish/Portuguese terms translated in English*	− *Patient‐centered medicine* (medicina centrada en la persona, 2) − *Patient‐centered care* (centralidad) − *Patient‐centered care* (centralidad en la atención) − *Person‐centered care* (cuidado centrado en las personas) − *Family and community health model/Integrity of care* (modelo de atención integral de salud familiar y comunitaria)	*6*	*Family‐centered care* (cuidado centrado na família)	1

*Note*: In one article (007) “patient‐centredness” and “patient‐centered care” were used interchangeably. In another article “patient‐centered care” and “patient‐centered orientation for health care” were used interchangeably. These two articles are thus represented twice in the table. In one article, “patient/family‐centered care” was used, as reported in the table. In eight of the articles on family‐centered care, the health context was either neonatal or pediatric care.

#### 3.2.1 Dimensions of the integrative model

Each dimension of the integrative model^5^ was covered in the selected literature. For an overview of the dimensions of the integrative model, see Supporting Information: Appendix S1. *Patient information* was the dimension that was covered most often, with 31 articles mentioning it. The dimension covered the least was *physical support*, with four articles mentioning it. For an overview of the frequency by which the dimensions were covered in the selected literature (see Table 2).

**Table 2:** Number of articles (*n* = 32) that covered the dimensions of the integrative model of patient‐centeredness ordered by frequency.

Dimension of the integrative model of patient‐centeredness	Count
Patient information	31
Patient involvement in care	26
Essential characteristics of the clinician	25
Clinician–patient relationship	25
Clinician–patient communication	24
Involvement of family and friends	23
Patient empowerment	21
Patient as a unique person	20
Coordination and continuity of care	19
Biopsychosocial perspective	17
Access to care	17
Emotional support	11
Integration of medical and nonmedical care	8
Teamwork and teambuilding	7
Physical support	4

In eight articles on FCC, the health context was either neonatal or pediatric care. In five of these articles, the covered dimensions of the integrative model were described as referring to the family, not only the patient. For example, in a study on FCC at neonatal intensive care units, the *clinician–patient relationship* naturally included the clinician–family relationship. Similarly, the family was included in the other dimensions. In the articles on PCC, the patient's family was referred to in a separate dimension, namely the *involvement of family and friends*, as done in the integrative model.

The dimensions *patient information* and *involvement of family and friends* were covered by all articles on FCC. However, the dimension *physical support* was not covered by any article on FCC. The dimensions *patient information, essential characteristics of the clinician, and patient involvement in care* were covered by over 80% of the articles on PCC. For a detailed overview of the frequency by which the dimensions were covered in articles on either PCC or FCC (see Table 3). For detailed overviews of the included literature and the coding of the dimensions of the integrative model, see Supporting Information: Appendices S2 and S3.

**Table 3:** Percentage of articles that included the dimensions of the integrative model of patient‐centeredness.

Main concept of the article	Patient‐centered care (*n* = 22)%	Family‐centered care (*n* = 10)%
Dimension of the integrative model of patient‐centeredness		
Essential characteristics of the clinician	86	60
Clinician–patient relationship	73	90
Patient as a unique person	73	40
Biopsychosocial perspective	68	20
Clinician–patient communication	72	80
Integration of medical and nonmedical care	32	10
Teamwork and teambuilding	23	20
Access to care	64	30
Coordination and continuity of care	64	50
Patient information	96	100
Patient involvement in care	82	80
Involvement of family and friends	59	100
Patient empowerment	73	50
Physical support	18	0
Emotional support	27	50

*Note*: Dimensions are ordered by original model and grouped by main concept of the articles. Percentages of minimum 80 are colored green, percentages of maximum 20 are colored red.

#### 3.2.2 Novel aspects of patient‐centeredness

In the literature on PCC, the following aspects were mentioned that are not explicitly covered by any dimension of the integrative model proposed by Scholl et al.^5^: “involvement of the local community,”(2) “patient as a multidisciplinary health care team member,” “acknowledgment of the family's potential.” In the literature on FCC, the following aspects were mentioned that are not covered by any dimension of the integrative model: “family as a care unit,”(9) “infrastructure to accommodate family members and to encourage their stay,”(2) “frequent reassessment of preferences as they may change over time.” These novel aspects could be used to extend distinct dimensions of the integrative model. However, we refrain from considering them aspects of PCC specific to Latin America.

#### 3.2.3 Concept analysis

We grouped the definitions of PCC and FCC into the following meaningful clusters: principles, activities, and results. Principles comprised autonomy, respect, collaboration, participation, and the form of care (coordinated and continuous). Activities included how PCC and FCC are implemented, for example, reviewing patient preferences, planning, evaluating, sharing information, and listening to the patient. As a result of the implementation of PCC and FCC, the impact on individuals and families stood out. For a complete overview of the concept analysis and the included definitions, see Supporting Information: Appendix S4.

#### 3.2.4 Patient‐centered care

The principles used in the definitions of PCC were dignity, respect, and participation. Autonomy and (co‐)responsibility were repeatedly named as well.

*PCC includes the following dimensions: biopsychosocial perspective; patient as a unique person; consideration of patient's values and beliefs; power and shared responsibility in care; therapeutic alliance to improve communication and participation in medical decision making; and the professional as a unique person*.^32^

The named activities for implementation were observing the patient's preferences, needs and values, sharing information, and improving the communication for the continuity of care. The suggested results of these activities were that patients and their families feel encouraged to make joint decisions about their care, as well as increased patient satisfaction and self‐management.

#### 3.2.5 Family‐centered care

The principles standing out in the definitions of FCC were dignity, respect and participation of the patient and family, and collaboration with them.

*The central assumptions of FCC are dignity and respect, in which professionals should be able to listen to patients and their families, have respect for the knowledge and beliefs of the patient and his/her family, because these assumptions are included in care, shared information, active participation and collaboration*.^60^

In addition, the family appeared as a subject of care and an essential source of support to the health care provider. The activity suggested for implementing FCC is sharing information with the family and the expected results are reduced anxiety and stress among the family members (Table 4).

**Table 4:** Principles, mechanisms, and results in PCC and FCC concepts.

Concept	Attributes	Mechanisms	Results
PCC	Respect (6) Dignity (3) Autonomy (4) Collaboration (3) Participation (2) Holistic framework (2) Continuous, articulated and quality provision of health services (2) Responsibility (2) Citizens' rights (1). Multidisciplinary, coordinated, continuous, and respectful care. Respectful, continuous, and coordinated care. Responsibility. Patient as subject. Coordination and integration of health care. Continuity of care. Coresponsibility (6). Patient and the professional as a unique person. Patient as an individual entity in a social environment. Citizen's rights. The role that the family plays in the development of health problems. Partnership. Centrality is interchangeable and their use may vary according to the context in which health services are provided.	Patient's preferences, needs, and values (7) Sharing information (6) Communication (3) Access to care (2) Individualization of care (2). Shared decisions making. Community collaboration. Cooperation and support at all levels of service provision. Patient's participation. Engage patients. Patient's needs, expectations, and preferences. Information and education. Involvement of family and friends in decision‐making. Consider users' and their families' needs. Therapeutic alliance. Relationships of trust. Meet the person holistically. Planning, delivery, and evaluation among patients, families, and providers.	Person‐centered health education and research. Person‐centered medical education and scientific health research. Patient satisfaction, decreased supportive care needs, and higher quality of life. PCC improves satisfaction and quality of life, reduces health care expenditures, and can reduce the supportive care needs of patients. Encourage patients and their families to make joint decisions about their own care. Preventative education. Patient‐centered model involves a two‐subject medicine model: the physician and patient. Improve the quality of the processes of care, reduce hospitalizations and emergency visits. Improve users' satisfaction and self‐management. Strengthen the doctor–patient relationship and make a realistic use of time and resources. PCC can improve health care utilization, efficiency, quality of care, and patient satisfaction. These attitudes and skills are, in fact, real tools that can help the person, through his own narrative, to reflect on his health–disease process.
FCC	Respect (6) Participation (6) Collaboration (4) Dignity (3) Partnership (2). Family as the basic unit of care. Family as essential source of support and main focus of attention. Autonomy. Family as the subject of care.	Sharing information (4). Planning, delivering, and evaluating health care. Listening between families. Community engagement. Implement services. Evaluate outcomes.	Opportunity for the family itself to define its own problems. Reduce anxiety of family members. Patient satisfaction. To reduce the stress that hospitalization. Give (to the family) some meaning to their own experience. Promote health and quality of life.

Abbreviations: FCC, family‐centered care; PCC, patient‐centered care.

#### 3.2.6 References given for the definitions of PCC and FCC

In the selected literature, 89 different references were used to define PCC and FCC. The references were published by authors from 18 countries and one by the World Health Organization. Of 60 references that were provided for the definition of PCC, 37 were international (25 from the United States of America), and 23 were from Latin America. A total of 15 of 23 Latin American references were from Brazil and almost all of these (14) were cited by authors from Brazil. A total of 4 of the 60 references were cited twice. The analysis of the references provided for the definition of FCC showed that of 29 references^19^ were international (eight from the United States of America), and 10 were from Latin America, more specifically from Brazil. Three references were cited twice. Also, one reference was cited once in a definition of PCC and once in a definition of FCC.

## 4 DISCUSSION

The aim of this study was to identify how PCC is conceptualized in Latin American countries. The analysis showed that two closely related but distinguishable concepts are discussed in the literature: PCC and FCC. Even though diverse terms were used to refer to PCC and FCC, an overlap between the provided definitions and the integrative model,^5^ and thus with international literature, was found. In most papers, international literature was cited to define PCC/FCC. There was little overlap in these citations, thus, no specific model of PCC was repeatedly used. Novel aspects not covered by the integrative model^5^ emerged as well. Most frequently mentioned was the identification of the family members as units of care.

Most dimensions of the integrative model^5^ were covered by at least two‐thirds of the included literature. Despite the reported differences to international literature, this shows that the conceptualization of PCC in Latin America considerably overlaps with the conceptualization in the global north. Regarding the dimensions described in the integrative model, we found that *sharing information* and *patient involvement* are most often mentioned in the literature. *Physical support*, *teamwork and teambuilding*, and *integration of medical and nonmedical care* were mentioned least. These results might reflect priorities but also the needs of the current health care systems in Latin America, as patients might continue to be placed at a passive role in their care.

Novel aspects not explicitly mentioned in the integrative model^5^ emerged from Latin American literature. The importance of infrastructure and the possibility for accommodation of family members were named. This is supposed to reduce anxiety and stress of family members. Latin America is a diverse region with different health care systems.^20^ The health care systems are built on postdictatorship neoliberal economic–political models, which explains that access and infrastructure are still not fully guaranteed in all the states of the region.^19^ Thus, in line with previous research, our results imply that health care infrastructure is one problem in Latin America that needs to be addressed to guarantee universal access to care and to enable a cascade of PCC activities.

The involvement of the local community has emerged as another novel aspect to the integrative model. In contrast to the involvement of the family, this aspect has not been explicitly stated in the international literature the integrative model is based on. Potential explanations are that community involvement is, dependent on the region, difficult to implement and thus less intuitive than involvement of families for example. Involvement of the local community has been proposed with the aim to make use of all given resources to improve the health care of individual patients. Another reason for the emergence of the local community as an aspect of PCC might be of historical nature. In Brazil, the Unified Health System was promoted by a health reform because there was a regionalized and decentralized network of health services, focusing on community participation.^64^ Other reasons might be a cultural imprint towards collectivism or a lack of resources.

The content analysis showed that the primary principles identified in PCC and FCC in Latin America are dignity, respect, and participation. These findings are in line with the conceptualization of PCC and FCC outside Latin America.^65^, ^66^ The overlap can be explained by the fact that the references used to define PCC and FCC were primarily from non‐Latin American countries, mainly from the United States of America. On the one hand, this is in line with the idea of a standard model of PCC. On the other hand, these results show that there is a scarceness of research groups specialized in PCC in Latin America, who have worked on proposing a conceptualization relevant for their own context, which is a contrast to North America, Europe, or Australia.

An international scoping review suggested FCC to be a part of PCC with a stronger focus on patient and family values, preferences, and needs.^67^ In contrast, our analysis showed differences between the concepts. Firstly, in the case of PCC, the focus was the patients themselves while the patient's family was referred to separately. The emphasis was placed on the co‐responsibility of the patient, excluding other significant actors such as relatives. In the definition of FCC, the focus was on the family and a collaboration established between the family and health professionals. This can be explained by the fact that the literature associates FCC with caring for children, elderly, or ailing individuals (not able to consent), thus, with the need for collaboration between family and HCPs.^68^ This focus can also be observed in the Latin American context. Secondly, we found differences in the activities and results of the two concepts. In PCC, the focus is on the encouragement of patients to take part in the decisions of their care, and the patients' satisfaction and self‐management. Contrary to PCC, for FCC the analysis showed that sharing information with the family is one of the most important activities aiming at the reduction of anxiety and stress of the family members, without necessarily enhancing an active involvement of the family members in the decision‐making process.

This article has some limitations. Firstly, following the recommendations of Peters,^33^ no quality appraisal of the included literature was conducted. However, there is literature arguing in favor of an assessment of quality in scoping reviews similarly to systematic reviews.^69^ Secondly, the gray literature search involved asking Latin American experts in PCC for relevant references. The experts were identified by the Latin American coauthors. This acquisition of experts might not have been exhaustive. In future studies, multiple independent researchers could be asked to achieve an exhaustive search of experts and thus of gray literature. Thirdly, numerous articles discussed PCC but failed to provide any meaningful definition of the concept. As we required an explanation of the concept for our concept analysis, we excluded these articles. Similarly, we excluded articles that only contained a keyword in the abstract but not in the title. Even though that was a considered decision, it may have caused a loss of information about the conceptualization of PCC in Latin America. Despite the limitation to articles with keywords in the title, the full‐text screening resulted in numerous articles missing a definition of PCC. Therefore, we propose that the scoping review provides a justifiably complete overview of the conceptualization of PCC in Latin American research and health policies.

Aside from the limitations, the scoping review offers distinctive strengths. The coauthors involved in the scoping review are experts in the field of PCC in Latin America and in Germany. The team jointly developed a search strategy that identifies as many sources on PCC as possible, even though the terminology in Latin America is diverse. Another strength is the identification of the conceptualization by use of a scoping review methodology. The method offers a broad overview of terminology and definitions and the opportunity to draw connections between present studies. Thus, this scoping review adds to previous research not covering studies from all over Latin America.^13^, ^27^

Our study shows that research on PCC is limited to a few Latin American countries. A strategy to support research on PCC in multiple countries in Latin America could be transnational studies on PCC, involving researchers and data from more than one country. The results also imply that future studies should clearly define the concept they aim to investigate. These strategies can foster the development of a common conceptualization of PCC. Future research can expand the present findings by assessing the needs of Latin American health care systems regarding PCC and barriers of its implementation. Novel aspects of PCC emerged from the present study. An integration of these novel aspects into the integrative model,^5^ either as new dimensions or as elements of existing dimensions, should be investigated in future empirical studies. The results also showed that few health ministries in Latin America have published documents discussing PCC, even though PCC is a declared aim. Thus, the concept should be defined and specific aims regarding PCC should be described in health care policies. The definition of PCC should be based on empirical research.

## 5 CONCLUSION

This scoping review synthesized and compared the conceptualization of PCC in 32 selected articles from Latin America published between 2006 and 2021. The analyses demonstrated a strong overlap between the integrative model and the definitions of PCC given in the literature. A conceptual distinction between PCC and FCC has been found. However, the results indicate a lack of standardization of the concept PCC in Latin America. The results will be used to develop a mixed‐methods study to understand the needs, barriers, and facilitators regarding PCC in Latin America. Based on the outcomes, the integrative model will be adapted to the Latin American context. The aim is to introduce a standard model for PCC that enables comparability of research, a transfer of outcomes between countries, and increasingly efficient communication on PCC in research, health policy, and clinical practice.

## AUTHOR CONTRIBUTIONS

All authors contributed to the conception and design of the study. Anne Klimesch, Alejandra Martinez‐Pereira carried out the searches, and the interpretation of the results. Anne Klimesch, Alejandra Martinez‐Pereira, and Cheyenne Topf were involved in the data extraction. All authors contributed to the writing up of the manuscript and approved the submitted version.

## CONFLICT OF INTEREST STATEMENT

Anne Klimesch, Alejandra Martinez‐Pereira, and Cheyenne Topf declare that there are no conflict of interest. Martin Härter, Isabelle Scholl, and Paulina Bravo declare that they currently are (Martin Härter, Paulina Bravo) or have been (Isabelle Scholl) members of the executive board of the International Shared Decision‐Making Society, which has the mission to foster the implementation of shared decision‐making and patient‐centered care. Paulina Bravo, Martin Härter, and Isabelle Scholl have no further conflict of interest.

## Supporting information

Supporting information.

## Data Availability

All data generated during this study was included in this published article.
