# Supplementary material for: Conceptualization of patient‐centered care in Latin America: A scoping review
Source: Health Expect. 2023 Jul 25;26(5):1820–31. doi: 10.1111/hex.13797 (PMC10485332; doi:10.1111/hex.13797)
Supplement: Supplementary file 1 — Supporting information. [file HEX-26--s001.docx]

**Appendix 1.** Integrative Model of Patient-Centeredness
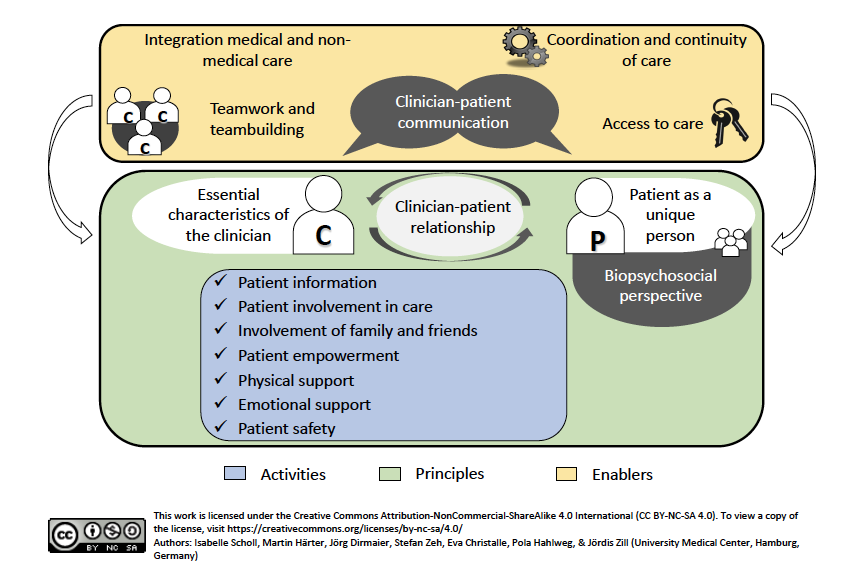
.

**Note.** The original model^1^ contains 15 dimensions. Here the extended model with 16 dimensions^2^ is shown.

References:

1. Scholl, I., Zill, J. M., Härter, M., & Dirmaier, J. (2014). An integrative model of patient-centeredness–a systematic review and concept analysis. PloS one, 9(9), e107828.

2. Zeh, S., Christalle, E., Hahlweg, P., Härter, M., & Scholl, I. (2019). Assessing the relevance and implementation of patient-centredness from the patients’ perspective in Germany: results of a Delphi study. BMJ open, 9(12), e031741

| **Appendix 2.** Coding for 15 Dimensions of the Integrative Model for Patient-Centeredness for the Articles on PCC Ordered by Year of Publication. | | | | | | | | | | | | | | | | | | | |
| --- | --- | --- | --- | --- | --- | --- | --- | --- | --- | --- | --- | --- | --- | --- | --- | --- | --- | --- | --- |
| **Title** | **Author(s)** | **Year** | **Country** | **Main concept** | **01** | **02** | **03** | **04** | **05** | **06** | **07** | **08** | **09** | **10** | **11** | **12** | **13** | **14** | **15** |
| Orientaciones para la implementación del modelo de atención integral de salud familiar y comunitaria | Ministerio de salud de Chile | 2013 | Chile | Principio centrado en las personas (Person-centered care) | X | X | X | X | X |  |  | X | X | X |  | X |  |  |  |
| Improving communication skill training in patient centered medical practice for enhancing rational use of laboratory tests: The core of bioinformation for leveraging stakeholder engagement in regulatory science | Moura, J. D. et al. | 2013 | Brazil | Patient-centered medical practice | X | X | X | X | X |  |  |  |  | X | X |  |  |  |  |
| Challenging Low Health Literacy in Rural Honduras: The Utilization of Charlas (Chats) for Patient-Centered Care | Sookhoo, L. | 2014 | Honduras | Patient-centered care / orientation for health care |  |  |  |  |  |  |  | X |  | X | X |  | X |  |  |
| Bases y perspectivas latinoamericanas sobre Medicina y Salud Centradas en la Persona | Wagner, P., et al. | 2015 | Latin America | Medicina centrada en la Persona (patient-centered medicine) | X | X | X | X | X |  | X |  | X | X |  | X |  |  |  |
| Using the clinical method of person-centered care in patients a System of Integral Attention to Health of the University of Veracruz | Dominguez, C. D. | 2015 | Mexico | Person-centered care |  | X | X |  |  |  |  |  |  | X | X |  | X |  |  |
| Patient centred care in interprofessional collaborative practice | Agreli, H. F., Peduzzi, M. & Silva, M. C. | 2016 | Brazil | Patient-centered care | X | X | X | X | X | X | X |  |  | X | X | X | X |  |  |
| Principios orientadores del Modelo Integral de salud familiar y comunitario desde la perspectiva de los usuarios | Dois, A., et al. | 2016 | Chile | Centralidad en la atención (patient-centered care) | X | X | X | X | X |  |  | X | X | X | X | X | X |  |  |
| Measurement of Family-centered care perception and parental stress in a neonatal unit | Balbino, F. S., Balieiro, M. M. F. G. & Mandetta, M. A. | 2016 | Brazil | Patient/family-centered care | X | X |  |  | X | X | X | X |  | X | X | X | X |  | X |
| Attributes of patient-centered primary care associated with the public perception of good healthcare quality in Brazil, Colombia, Mexico and El Salvador | Doubova, S. V. et al. | 2016 | Latin America | Patient-centred care | X | X | X | X | X | X | X | X | X | X | X | X | X | X | X |
| Patient-centred care and shared decision making in Chile: Rising momentum for progress and implementation in clinical practice | Bravo, P. et al. | 2017 | Chile | Patient-centred care | X | X | X | X | X |  |  | X |  | X | X | X | X |  |  |
| A comprehensive care program achieves high remission rates in rheumatoid arthritis in a middle-income setting. Experience of a Center of Excellence in Colombia | Santos-Moreno, P. et al. | 2017 | Colombia | Patient-centred care | X |  | X |  |  | X |  | X | X | X | X |  | X | X |  |
| Atributos y características de los principios orientadores del Modelo de Atención Integral de Salud Familiar y Comunitaria desde la perspectiva de expertos en APS | Dois, A., Bravo, P. & Soto, G. | 2017 | Chile | Family and community health model (Integrity of care) | X | X | X | X |  |  |  | X | X | X | X | X | X |  | X |
| Patient-centered primary care and self-rated health in 6 Latin American and Caribbean countries: Analysis of a public opinion cross-sectional survey | Guanais, F., Doubova, S. V., Leslie, H. H., et al. | 2018 | Latin America | Patient-centered primary care | X |  |  |  | X |  |  | X | X | X |  |  | X |  |  |
| Participación de los usuarios en las decisiones clínicas en la atención primaria de salud en Chile | Bravo, P. et al. | 2018 | Chile | Person-centered care |  | X | X |  | X |  |  |  | X | X | X |  | X |  |  |
| Approaching Spirituality Using the Patient-Centered Clinical Method | de Oliveira, J. A. C., Anderson, M. I. P., Lucchetti, G., et al. | 2019 | Brazil | Patient-centered clinical method | X | X | X | X | X | X |  |  |  | X | X | X | X |  | X |
| Perspectivas latinoamericanas sobre medicina centrada en la persona | Perales, A. et al. | 2019 | Latin America | Medicina centrada en la persona (patient-centered medicine) | X | X | X | X | X |  |  |  | X | X | X | X | X |  |  |
| Agenda para a pesquisa sobre o cuidado centrado no paciente no Brasil | Rodrigues, J. L. S. Q., Crisóstomo Portela, M. & Malik, A.M. | 2019 | Brazil | Person-centered care | X | X |  |  | X |  |  |  | X |  |  |  |  |  |  |
| Psychometric validation of a Patient-Centred Quality of Cancer Care Questionnaire in Mexico | Doubova, S. V. et al. | 2020 | Mexico | Patient- centredness & patient-centred care | X |  | X | X | X | X |  | X | X | X | X | X | X | X | X |
| Association of supportive care needs and quality of patient‐centered cancer care with depression in women with breast and cervical cancer in mexico | Doubova, S. V. & Pérez‐Cuevas, R. | 2020 | Mexico | Patient-centered care | X |  |  | X |  |  |  | X | X | X | X |  |  |  |  |
| Validation of hospital effectiveness indicators in the patient-centered care dimension | Soares Seiffert, L. et al. | 2020 | Brazil | Patient-centered care | X | X | X | X | X |  |  | X |  | X | X | X | X |  |  |
| Social inequalities in supportive care needs and quality of patient-centered care of cancer patients in Mexico | Doubova, S. V. et al. | 2021 | Mexico | Patient-centered care | X |  |  | X |  |  |  | X | X | X | X |  |  |  |  |
| Marco operativo estrategia de cuidado integral centrado en las personas para la promoción, prevención y manejo de la cronicidad en contexto de multimorbilidad | Ministerio de salud de Chile | 2021 | Chile | Cuidado centrado en las personas (Person-centered care) | X | X | X | X | X | X | X | X | X | X | X | X | X | X | X |
| *Note.* Dimensions of the integrative model of patient-centeredness and the assigned numbers: [1] essential characteristics of the clinician, [2] clinician-patient relationship, [3] patient as a unique person, [4] biopsychosocial perspective, [5] clinician-patient communication, [6] integration of medical and non-medical care, [7] teamwork and teambuilding, [8] access to care, [9] coordination and continuity of care, [10] patient information, [11] patient involvement in care, [12] involvement of family and friends, [13] patient empowerment, [14] physical support, [15] emotional support. | | | | | | | | | | | | | | | | | | | |

| **Appendix 3.** Coding for 15 Dimensions of the Integrative Model for Patient-Centeredness for the Articles on FCC Ordered by Year of Publication. | | | | | | | | | | | | | | | | | | | |
| --- | --- | --- | --- | --- | --- | --- | --- | --- | --- | --- | --- | --- | --- | --- | --- | --- | --- | --- | --- |
| **Title** | **Author(s)** | **Year** | **Country** | **Main concept** | **01** | **02** | **03** | **04** | **05** | **06** | **07** | **08** | **09** | **10** | **11** | **12** | **13** | **14** | **15** |
| Family-centered care from the perspective of nurses in the Neonatal Intensive Care Unit | Gomes da Silva, T. R. et al. | 2006 | Brazil | Family-centered care |  | X |  |  | X |  |  |  | X | X | X | X | X |  |  |
| Medical decision making: paternalism versus patient-centered (autonomous) care | Rodriguez-Osorio, C. A., & Dominguez-Cherit, G. | 2008 | Mexico | Family-centered care |  | X |  |  | X |  |  |  |  | X | X | X |  |  | X |
| Family-centered care in the context of children with disabilities and their families: A reflective Review | Barbosa, M. A. M., Balieiro, M. & Pettengill, M. A. M. | 2012 | Brazil | Family-centered care | X | X | X |  |  | X |  | X | X | X | X | X | X |  | X |
| Use of narrative as an awareness strategy for a Family-Centered Care model | Amador, D. D. et al. | 2015 | Brazil | Family-centered care model | X |  |  | X | X |  |  |  |  | X | X | X |  |  |  |
| Family-centeredness and community orientation according to three child health care models | Carvalho de Brito Santos, N. C. et al. | 2016 | Brazil | Family-centeredness |  | X |  |  | X |  |  | X | X | X |  | X | X |  |  |
| Family-centered care in a pediatric intensive care unit: professionals' perceptions | de Andrade Sampaio, A. et al. | 2017 | Brazil | Family-centered care |  | X |  |  |  |  |  |  |  | X |  | X |  |  |  |
| Family-centered care in neonatal and pediatric intensive care unit: nurse's vision | Felipin, L. C. S., Merino, M. & Baena, J. A. | 2018 | Brazil | Family-centered care | X | X | X |  | X |  |  |  |  | X | X | X | X |  | X |
| Cuidado centrado na família e sua aplicação na enfermagem pediátrica | Peres PintoI, J. et al. | 2019 | Brazil | Family-centered care | X | X | X | X | X |  | X |  |  | X | X | X |  |  | X |
| Family-centered care in neonatology: health workers’ and families’ perceptions | Borotta Uema, R. T. et al. | 2020 | Brazil | Family-centered care | X | X |  |  | X |  |  | X | X | X | X | X | X |  | X |
| Family-Centered practice in a Brazilian rehabilitation network service | Antunes, A. A. M. & Vaz, D. V. | 2021 | Brazil | Family-centered practice | X | X | X |  | X |  | X |  | X | X | X | X |  |  |  |
| *Note.* Dimensions of the integrative model of patient-centeredness and the assigned numbers: [1] essential characteristics of the clinician, [2] clinician-patient relationship, [3] patient as a unique person, [4] biopsychosocial perspective, [5] clinician-patient communication, [6] integration of medical and non-medical care, [7] teamwork and teambuilding, [8] access to care, [9] coordination and continuity of care, [10] patient information, [11] patient involvement in care, [12] involvement of family and friends, [13] patient empowerment, [14] physical support, [15] emotional support. | | | | | | | | | | | | | | | | | | | |

**Appendix 4.** Concept Analysis.

| Author(s) | Year | Country | Main concept | Definition/description of main concept | Attributes | Mechanisms | Results |
| --- | --- | --- | --- | --- | --- | --- | --- |
| Barbosa, et al. | 2012 | Brazil | Family-centered care | FCC is an approach that acknowledges the importance of the family as a recipient of care, ensuring the participation of all its members in the planning of actions and revealing a new model of care, offering the opportunity for the family itself to define its own problems. Respecting the child’s individuality and that of his/her family is decisive and represents a permanent challenge for health services and health workers, as well. It requires the staff to be open and attentive to the interactions and impact of experiences and also to provide knowledge concerning the dynamics, beliefs, and ways families can adapt to different situations. | Respect  Partnership  Participation |  | Opportunity for the family itself to define its own problems |
| Segantini Felipin, et al. | 2018 | Brazil | Family-centered care | Family-Centered Care is a philosophy that recognizes the family as a fundamental part of health care and aims to stimulate the family’s bond with the patient, ensuring their participation in the planning of health actions. Patient and family-centered care is an approach to planning, delivering, and evaluating health care that builds on mutually beneficial partnerships between professionals, patients, and families in a way that all involved are recognized as care recipients, regardless of their age, reducing not only the anxiety of family members, but also increasing patient satisfaction with care. The CCF concept also aims to define the quality of care provided according to the patients and their families vision and to promote the discussion of patient autonomy in relation to their health needs. The central assumptions of CCF are dignity and respect, in which professionals should be able to listen to patients and their families, have respect for the knowledge and beliefs of the patient and his/her family, because these assumptions are included in care, shared information, active participation and collaboration. | Dignity  Respect  Participation  Collaboration | Planning, delivering, and evaluating health care | Reduce anxiety of family members  Patient satisfaction |
| Borotta Uema, et al. | 2020 | Brazil | Family-centered care | There is a paradigm shift in the context of health care, where the focus, previously centered on curativism, today assumes a much broader role, bringing care centered on the patient and their family into the hospital environment, a moment in which humanization of care and quality of life become a priority, decreasing the specific attention to the illness process. This new approach is translated as Family-Centered Care (FCC), whose characteristic encompasses a philosophy of care provided to the patient and their family, giving voice to both. This model uses assumptions that must be followed throughout the hospitalization, whether in the neonatal, pediatric, or adult contexts. The main ones include the application of: dignity and respect (the health professionals respect the choices, values and beliefs of the patient and family); shared information (the professionals communicate and share information completely and impartially with patients and families); participation (patients and families are encouraged and supported to participate in decision-making); and collaboration (patients and families are included and understood as the support base of the institution, assisting in the development of policies and programs, in professional education and in the provision of care). | Dignity  Respect  Participation  Collaboration | Sharing  information |  |
| Gomes da Silva, et al. | 2006 | Brazil | Family-centered care | Family-centered Care is a care model where the family is inserted in the planning and decision-making of care along with health professionals and the patient. The family is aware of the potential benefits and risks and is granted comfort regarding the assistance given and receives the assurance of its quality. Family-centered care follows the principles established in the agreement between relevant institutions that guide the care practice, considering the sharing of information among all involved in the care in an impartial, clear and objective way, respecting the values and wishes of the patient and encouraging this and the family to have an active participation in the planning of treatment. Therefore, in family-centered care, the family is acknowledged as a partner of the team and its participation in the assistance contributes to the affective bonding between caregiver and patient, helping to reduce the stress that hospitalization and continuity of care at home after discharge causes to parents. It is noteworthy that this model considers the family as the basic unit of care, inseparable from the patient, especially when this is a child. It is the responsibility of the health team, as a whole, to include caregivers in the caring activities, involving them effectively in the moment of planning these activities, with basis to keep them aware of their role and their importance in the group, not simply as performers of procedures providing care with focus in the biological process of the disease that affects the patient. | Partnership  Family as the basic unit of care | Sharing information | To reduce the stress that hospitalization |
| de Andrade Sampaio, et al | 2017 | Brazil | Family-centered care | The model of care centered on the patient and the family is an innovative and effective strategy in establishing a partnership between health professionals, family, and child. It is internationally recognized for being a model of care in which the family is an essential source of support and the main focus of attention is not the disease but the individual and his family. It is based on the effective sharing of information, on unrestricted access to the child, on the effective participation of parents in decision-making, and on mutual respect. | Respect  Partnership  Participation  The family is an essential source of support and the main focus of attention | Sharing  information |  |
| Rodriguez-Osorio, C. A., & Dominguez-Cherit, G. | 2008 | Mexico | Family-centered care | Although shared decision making and patient autonomy are desirable, the situation may be different when considering critically ill patients. These patients are often incapable of providing consent, and unless they had previously filed to the contrary, their families receive full information on their diagnosis, prognosis, and treatment, and are anticipated to make medical decisions on their behalf. For this reason, healthcare providers in ICU have devoted considerable attention to families, creating the concept of family-centered care. | Autonomy | Sharing  information |  |
| Amador, et al. | 2015 | Brazil | Family-centered care model | The Family-Centered Care (FCC) model (...) is based on four concepts: dignity and respect, shared information, participation, and collaboration, mediated by continued negotiation between the healthcare team and the patients´ families. Inviting families to narrate their experiences is a way of helping them give some meaning to their own experience. Listening to other people’s narrative can be considered a reliable way of gaining access to their personal lives and provides a powerful resource to learn about the human experience. Narrative is included in the living process and it is a spontaneous activity insofar as it is delivered in an appropriate environment. | Dignity  Respect  Collaboration  Participation | Sharing  information  Listening between families | Give (to the family) some meaning to their own experience |
| Carvalho de Brito Santos, et al. | 2016 | Brazil | Family-centeredness | The family-centeredness considers the family as the subject of care, with potential for care. In the community orientation, the families’ needs are recognized in function of the geographical and socio-economic-cultural context they live in, besides its importance to assess the health services. In each attribute, dimensions are assessed that are important for the integrality of care delivered to individuals, families and communities. In the family-centeredness, the items are related to the professionals’ concern with what the child’s family thinks about the treatment and the care provided, the concern with existing problems in the family, meeting with other members if the relative thinks that is necessary. What the community orientation is concerned, the dimensions include a team member making a home visit, the service’s engagement in the community’s health problems through household surveys and the invitation of family members to participate in the health council. | The family as the subject of care  Participation | Community engagement |  |
| Antunes, A. A. M. & Vaz, D. V. | 2021 | Brazil | Family-centered practice | FCP is organized around three core principles: (1) respect for children and their families, (2) appreciation of the family’s impact on the child’s well-being, and (3) collaboration between families and service providers. In FCP, families and service providers collaborate to identify goals and priorities, plan and implement services, and evaluate outcomes. FCP can improve service efficiency to promote health and quality of life | Respect  Collaboration | Implement services  Evaluate outcomes | Promote health and quality of life |
| Wagner, P., et al. | 2015 | Latinoamérica | Medicina Centrada en la Persona (patient-centered medicine) | Key concepts: ethical commitment, holistic framework, relational emphasis, cultural sensitivity, individualization of care, diagnostic understanding and shared clinical decisions among clinicians, patient and family, health service planning in partnership with the community, and development of person-centered health education and research. | Holistic framework | Individualization of care  Shared decisions making  Community collaboration | Person-centered health education and research. |
| Perales, et al. | 2019 | Perú, Colombia, Chile & Uruguay | Medicina centrada en la persona (patient-centered medicine) | Eight principles of person-centered medicine (PCM) (1): ethical commitment, holistic framework, cultural sensitivity, and responsiveness; communicative and relational focus; individualization of the clinical care program; establishing common ground between clinicians, patient, and family for understanding and action; organization of integrated, person- and community-centered services; person-centered medical education and scientific health research. | Holistic framework | Individualization of care  Communication | Person-centered medical education and scientific health research. |
| Balbino, et al. | 2016 | Brazil | Patient/family-centered care | The Patient and Family-Centered Care (PFCC) Model has been proposed as an innovative approach to the planning, provision and health evaluation, conducted mutually by the partnership between health care providers, patients and families. It can be applied to patients of all ages and practiced in any health facility. In the neonatal context, studies show that the PFCC is becoming the standard of care in the world, where the family is understood as a primary source of strength and support of the newborn. This perspective of care incorporates concepts such as unrestricted access to the child, respect, information, choice, service flexibility, autonomy of the involved subjects, cooperation and support at all levels of service provision. | Respect  Autonomy | Sharing information  Cooperation and support at all levels of service provision |  |
| Agreli, et al. | 2016 | Brazil | Patient-centered care | These (Brazilian) studies focused on PCC in connection with the following aspects: 1) Enlarged healthcare perspective, 2) Patients’ participation in care and 3) Humane care. [...] Patients’ participation in care is defined as an opportunity for the exercise of citizens’ rights in the quest for autonomy (related to ideas of freedom, leading role,respect for subjectivity) as an important condition for self-care. | Citizens’ rights  Autonomy | Patients’ participation |  |
| Doubova, et al. | 2020 | Mexico | Patient-centered care | Patient‐centered cancer care aims to engage patients in their healthcare. It promotes multidisciplinary, coordinated, continuous, and respectful care, and the sharing of information for decision‐ making. It is associated with higher patient satisfaction, decreased supportive care needs, and higher quality of life. | Multidisciplinary, coordinated, continuous, and respectful care | Engage patients  Sharing  information | Patient satisfaction, decreased supportive care needs, and higher quality of life. |
| Doubova, et al. | 2021 | Mexico | Patient-centered care | Patient-centered care (PCC) aims at meeting needs, expectations, and preferences of patients through respectful, continuous, and coordinated care. Moreover, PCC improves satisfaction and quality of life, reduces healthcare expenditures, and can reduce the supportive care needs of patients. | Respectful, continuous, and coordinated care | Patient’s preferences, needs, and values | PCC improves satisfaction and quality of life, reduces healthcare expenditures, and can reduce the supportive care needs of patients. |
| Soares Seiffert, et al. | 2020 | Brazil | Patient-centered care | For understanding and delivering patient-centered care, the American Institute for Patient- and Family-Centered Care considers the following core concepts: dignity and respect, information sharing, participation, and collaboration. These concepts demonstrate that valuing their perspectives, values, beliefs, cultural backgrounds, and choices improves health care (DeRosa et al., 2019). In this way, receiving complete and unbiased information, as well as sharing it with health professionals, encourage patients and their families to make joint decisions about their own care. | Dignity  Respect  Collaboration  Participation | Patient’s preferences, needs, and values  Sharing information | Encourage patients and their families to make joint decisions about their own care. |
| Sookhoo, L. | 2014 | Honduras | Patient-centered care / patient-centered orientation for health care | A patient-centered orientation for health care includes not only appropriate messages, collaborative goal-setting, and delivering post-visit reinforcements but also a redesign of the current care model, which mainly consists of periodic treatment (Paasche-Orlow et al. 2006).  Providing medical care with a patient-centered orientation should include the responsibility to provide preventative education in some form. | Responsability  Collaboration |  | Preventative education |
| Moura, et al. | 2013 | Brazil | Biomedical model of care | Five key dimensions can be recognized to differentiate the practice of clinical care focused on the person of the biomedical model of care as part of the Biomedical Model. 1) Biopsychosocial perspective – addresses the patients’ triaxial diagnostic process, and considers the importance of opening a "hidden agenda" for the patient, where they take care of a dysfunctional state and not just a physical illness; 2) Patients as a subject – considers the patients’ biography as well as their individual experience with the disease. “You must understand the signs and symptoms found by the physician and patient dissatisfaction, shown by and a manifestation of their individual conflicts and problems”; 3) Shared strengths and responsibilities – the conflict between the medical and patient autonomy is central to the physician-patient relationship; 4) Therapeutic alliance – the doctor must act so that the patient perceives the relevance and effect of the proposed treatment and agree to the objectives outlined, always considering the affective and cognitive characteristics of the patient; 5) Physician as a subject – The biomedical model focuses on the diagnosis and treatment, based on the "medicine of a subject”, i.e., the physician. On the other hand, the patient-centered model involves a two-subject medicine model: the physician and patient. | Patients as a subject  Responsability  Autonomy |  | Patient-centered model involves a two-subject medicine model: the physician and patient |
| Guanais, et al | 2018 | Brazil, Colombia, El Salvador, Jamaica, Mexico, and Panama | Patient-centered primary care | Patient-centered healthcare has emerged as a person-oriented model of care aimed at meeting population needs, expectations, and preferences. Studies from the United States and the United Kingdom have shown the positive effect of patient-centered healthcare in improving the quality of the processes of care, reducing hospitalizations and emergency visits (and consequently healthcare costs), and improving users’ satisfaction and self-management. Within PC, a number of patient-centered healthcare attributes have been shown to be associated with perception of good healthcare quality, such as the availability of a PC provider who “knows relevant information about a patient’s medical history,” “solves most of the health problems,” “spends enough time with the patient,” “coordinates healthcare,” and “is easy to communicate with”. |  | Patient’s needs, expectations, and preferences | Improve quality of the processes of care, reduce hospitalizations and emergency visits  Improve users’ satisfaction and self-management |
| Doubova, et al. | 2016 | Brazil, Colombia, Mexico and El Salvador | Patient-centred care | The Picker-Commonwealth Program has proposed a comprehensive PCC model that takes into account the experiences of patients receiving healthcare across eight do- mains: (1) respect for patient-centered values, preferences and needs; (2) coordination and integration of healthcare across services; (3) information and education of patients to facilitate autonomy and self-care; (4) physical comfort; (5) emotional support; (6) involvement of family and friends in decision-making; (7) continuity of care; and (8) easy access to care (Shaller 2007). | Respect  Autonomy  Coordination and integration of healthcare  Continuity of care | Information and education  Involvement of family and friends in decision-making  Access to care |  |
| Bravo, et al. | 2017 | Chile | Patient-centred care | Patient-centred care (PCC) was defined by the Ministry of Health in 2013. According to this definition, PCC must consider users’ and their families needs, so they can be prioritised and addressed by adopting a patient-professional relationship that is based on co-responsibility of care. PCC includes the following dimensions: biopsychosocial perspective; patient as a unique person; consideration of patient’s values and beliefs; power and shared responsibility in care; therapeutic alliance to improve communication and participation in medical decision making; and the professional as a unique person. | Co-responsability  Patient and the professional as a unique person | Improve communication  Consider users’ and their families needs  Therapeutic alliance |  |
| Santos-Moreno, et al. | 2017 | Colombia | Patient-centred care | A PCC model, as defined by the United States Medical Institute, is one that provides respectful care, answering the patient’s individual preferences, needs, and values, ensuring that these guide all clinical decisions. The philosophy behind a PCC model is the widespread respect for the patient as an individual entity in a social environment, which implies that they must be treated as individuals who need to be seen as multidisciplinary healthcare team members to ensure their considerations and requirements in health are taken into account. | Respect  Patient as an individual entity in a social environment | Patient’s preferences, needs, and values |  |
| Ministerio de salud de Chile | 2013 | Chile | principio centrado en las personas (Person-centered care) | The needs and expectations of individuals and their families are considered from their particular way of defining and prioritizing them, for which a relationship model must be established between the health team, the individuals, their families and the community, based on co-responsibility for health care and emphasizing the rights and duties of the individuals, both the health team and the users. The most relevant objectives related to this principle are: - To establish a treatment of excellence to people at any point of contact, based on effective communication and respect for the dignity and people’s rights. - Establish relationships of trust and assistance. - Facilitate the population's access to health care. - To ensure access to care for the most vulnerable groups. | Respect  Dignity  Co-responsability  Citizen’s rights | Effective communication  Relationships of trust  Access to care |  |
| Dois, et al. | 2016 | Chile | Centralidad en la atención (patient-centered care) | The centrality -centeredness- considers the co-responsibility between the professional team, the people (their families) and the community, people (their families) and the community. Emphasizes the rights and duties of the users involved and focuses and focuses attention on the person and their needs, providing them with and their needs, providing them with the necessary information necessary to participate in decisions related to their care. | Co-responsability | Patient’s preferences, needs, and values  Sharing information |  |
| Dominguez, C. D. | 2015 | Mexico | Person-centered care | The person-centered approach includes subjective aspects in the physician-patient relationship, the human experience of illness, the individual characteristics of each person and considered a shared responsibility, which six aspects must be taken on account: exploring the disease and ailment , meet the person holistically, finding a common ground with handling problems, do health promotion, strengthen the doctor-patient relationship and make a realistic use of time and resources. | Co-responsibility | Meet the person holistically | Strengthen the doctor-patient relationship and make a realistic use of time and resources. |
| Doubova, et al. | 2020 | Mexico | Patient- Centredness / patient-centred care | Patient-centredness is a core component of high-quality healthcare. It calls on providers to respect patients’ values and preferences, facilitate access to continuous and coordinated care, address patients’ physical and emotional needs, and provide adequate information to enable patient participation in healthcare-related decision-making and self-care. Patient-centred care can improve healthcare utilisation, efficiency, quality of care and patient satisfaction | Respect | Patient’s preferences, needs, and values  Sharing information | Patient-centred care can improve healthcare utilisation, efficiency, quality of care and patient satisfaction |
| de Oliveira, et al. | 2019 | Brazil | Patient-centered clinical method | The PCCM is an example of a clinical tool that can help to promote biopsychosocial– spiritual or integral care. This methodology had its origin based on investigations that indicated that the people were looking for a medical service that values the motivations for the consultation; seeks to understand their world (including emotional and existential issues); and values the physician–patient relationship, the shared decision and the prevention and the promotion of health.[...]. In patient-centered practice, the health professional should use open-ended questions, comprehensive listening and a non-critical stance. These attitudes and skills are, in fact, real tools that can help the person, through his own narrative, to reflect on his health–disease process. Therefore, help people to revise and give a new meaning to the complex factors related to their health problem. | Co-responsability | Patient’s preferences, needs, and values | These attitudes and skills are, in fact, real tools that can help the person, through his own narrative, to reflect on his health–disease process |
| Bravo, et al. | 2018 | Chile | Person-centered care | Person-centered care (PCC) considers that the user is co-responsible for his or her care, in collaboration with the health care team. Therefore, users need to receive adequate information to enable them to users need to receive adequate information to enable them to participate actively and in an informed manner in the decisions related to their care and to feel care and feel that they are treated in a dignified with dignity | Co-responsability  Dignity  Collaboration  Participation | Sharing information |  |
| Dois, A., Bravo, P. & Soto, G. | 2017 | Chile | Family and Community Health Model (Integrity of care) | Integrity of care considers the continuous, articulated and quality provision of a wide range of services throughout the life cycle, for individuals, their families and the community in which they live. In addition, it incorporates the belief system associated with health problems and their and their treatment, and the role that the family plays in the development of health problems. | The role that the family plays in the development of health problems  Continuous, articulated and quality provision of health services  . |  |  |
| Ministerio de salud de Chile | 2021 | Chile | Cuidado centrado en las personas (Person-centered care) | Comprehensive care centered on the person, from the Comprehensive Family and Community Health Care Model is understood as the consideration of biological, psychological, social, and spiritual dimensions in all stages of the life course process and in the relevance of the state of health-disease. Integrality, from the perspective of the multidimensional understanding of people's problems, reveals the system of beliefs and meanings that people give to health problems and socially acceptable therapies. These are addressed through comprehensive, consensual and continuous care plans coordinating health benefits. | Continuous, articulated and quality provision of health services |  |  |
| Peres PintoI, et al. | 2019 | Brazil | Family-centered care | This approach to health care as a partnership-based, mutually beneficial process of planning, delivery, and evaluation among patients, families, and providers. It is care for patients of all ages and can be practiced in any health care setting, by all health care professionals. | Partnership | Planning, delivery, and evaluation among patients, families, and providers |  |
| da Silva de Queiroz Rodrigues, et al | 2019 | Brazil | Person-centered care | Patient-centered care is defined as the provision of care in a respectful manner that is responsive to the needs, preferences, and values of the person being cared for and ensures that these values guide all clinical decisions. Today, a variety of terms convey the centrality of health care to the subject. These terms are interchangeable and their use may vary according to the context in which health services are provided. | Centrality is interchangeable and their use may vary according to the context in which health services are provided. | Patient’s preferences, needs, and values |  |
